# SNP Mining in Functional Genes from Nonmodel Species by Next-Generation Sequencing: A Case of Flowering, Pre-Harvest Sprouting, and Dehydration Resistant Genes in Wheat

**DOI:** 10.1155/2016/3524908

**Published:** 2016-03-14

**Authors:** Zhong-Xu Chen, Mei Deng, Ji-Rui Wang

**Affiliations:** Triticeae Research Institute, Sichuan Agricultural University, Chengdu 611130, China

## Abstract

As plenty of nonmodel plants are without genomic sequences, the combination of molecular technologies and the next generation sequencing (NGS) platform has led to a new approach to study the genetic variations of these plants. Software GATK, SOAPsnp, samtools, and others are often used to deal with the NGS data. In this study, BLAST was applied to call SNPs from 16 mixed functional gene's sequence data of polyploidy wheat. In total 1.2 million reads were obtained with the average of 7500 reads per genes. To get accurate information, 390,992 pair reads were successfully assembled before aligning to those functional genes. Standalone BLAST tools were used to map assembled sequence to functional genes, respectively. Polynomial fitting was applied to find the suitable minor allele frequency (MAF) threshold at 6% for assembled reads of each functional gene. SNPs accuracy form assembled reads, pretrimmed reads, and original reads were compared, which declared that SNPs mined from the assembled reads were more reliable than others. It was also demonstrated that mixed samples' NGS sequences and then analysis by BLAST were an effective, low-cost, and accurate way to mine SNPs for nonmodel species. Assembled reads and polynomial fitting threshold were recommended for more accurate SNPs target.

## 1. Background

Next-generation sequencing technologies (e.g., Solexa/Illumina, SOLiD/ABI, 454/Roche, and HeliScope/Helicos) have opened a new field to genotype analysis of nonmodel organisms. Technologies generating long-sequence reads (200–400 bp) are increasingly used in evolutionary studies of nonmodel organisms, but the short sequence reads (30–150 bp) can be produced at lower cost and with more sequence data per run [1]. Short-read technologies are typically best suitable for resequencing projects where a reference genome on which the reads can be mapped is already available [2, 3]. Because of the short-read lengths, the application of NGS technologies has generally been restricted to nonmodel organisms for which the genome sequences is not available. However, recently many algorithmic and experimental advances have made it possible to succeed at de novo sequence projects [4–6]. But some of these reads (both long and short) contain adapters and other exogenous contents by experimental designs. On other cases, adapters were sequenced inadvertently when they are out of operational errors and other unknown reasons. If these adapters were not trimmed out, they would interfere with the downstream data analysis, such as mapping the reads to the reference genome and de novo assembly [7, 8].

For most of the next-generation sequencing technologies (both single-read and paired-end libraries), the quality of the sequencing gets lower while approaching the end of the reads. If excessive sequencing errors occurred in the end of the reads, this would affect the accuracy of mapping and other downstream analysis, even if the reads contain high-quality bases in the beginning. To prevent otherwise high-quality reads from being rejected during quality filtering or from influencing mapping or assembly processes, it can be beneficial to trim bases from poor-quality ends of reads [9]. Illumina's sequencing by synthesis (SBS) technology (Illumina report) is the most successful and widely adopted next-generation sequencing platform worldwide, which is also the only platform that offers a short-insert paired-end capability for high-resolution genome sequencing as well as long-insert paired-end reads using the same robust chemistry for efficient sequence assembly, de novo sequencing, large-scale structural variation detection, and so on [10].

**Figure 1:**
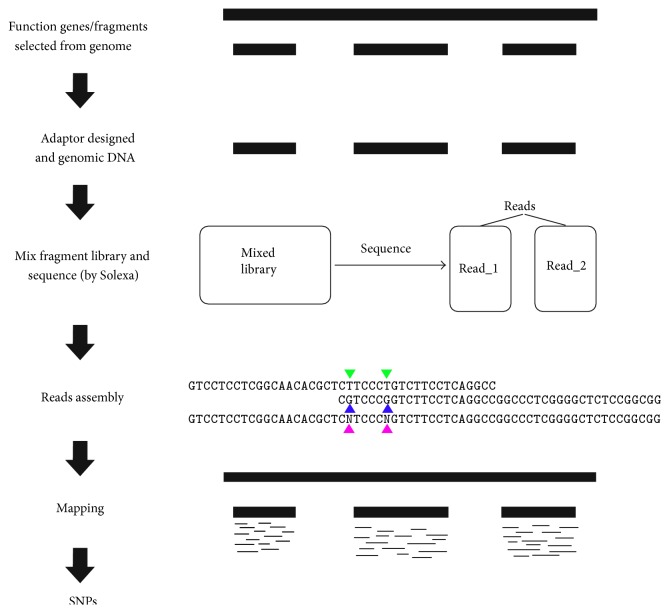
The main steps to mine SNPs on function genes.

Triticeae has large and complex genomes with a great abundance of repeated sequences, which does not have a very good whole genome reference available now. Studies on these plants whose polyploidy has further increased genome size and complexity have not been able to fully take advantage of next-generation sequencing for SNP discovery (since SNPs are of more importance on functional genes coding region, 16 genes were molecular-cloned and resequenced form wheat as a case). After these genes were cloned and mixed, these genes were resequenced by NGS Solexa platform and SNPs were called following our pipelines in Figure 1. The polynomial fitting equation was applied to find the best threshold value to filter the low quality SNPs.

## 2. Materials and Methods

### 2.1. DNA Isolation and PCR Amplification

Genomic DNA was extracted from leaves of single plants of about 2 weeks old with a modified CTAB protocol. 16 functional genes were randomly selected from NCBI database with the sequences as reference in the following study (Table 1).

Anchored primers were designed on the basis of conserved sequences outside of the polymorphic regions. PCR amplification was performed with GeneAmp PTC-240 cycler (Bio-Rad) in 50 *μ*L volume which consisted of 100 ng of genomic DNA, 100 *μ*M of each dNTP, 1 *μ*M of each primer, 1 U Taq polymerase with high fidelity, 1.5 mM Mg^2+^, and 1x PCR buffer. The cycling parameters were 95°C for 5 min to predenature, followed by 35 cycles of 95°C for 50 sec, 50–60°C for 30 sec and 72°C 45 sec, and a final extension at 72°C for 5 min. Desired PCR products were obtained by agarose gel. The fragments of genes were mixed with similar concentration.

### 2.2. Sequence Data Quantity and Quality

**Figure 2:**
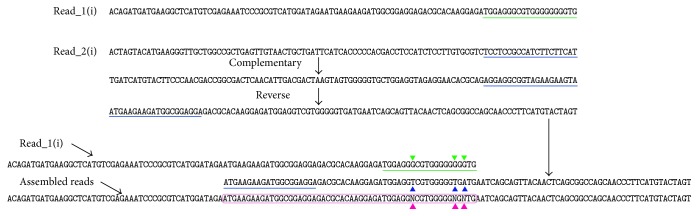
Reads assembly. A(i).fastq and B(i).fastq were one-paired-end reads. The color lines were low quality parts (20 bp). Purple wireframe was the assembled reads part. Solid triangle was the locus which was not consistent in two reads. Paired-end reads were reverse compliment reads. To assemble the two reads, reverse compliment sequence should be calculated by one of them and the other one should be kept. The entire mismatch locus would be set as “N.”

Ten mixed DNA samples were sequenced in one run with Illumina Solexa platform. We get the sequencing result as pairing reads, which was stored in two fastq files, “read_1.fq” and “read_2.fq,” respectively. The sequences at the same position from read_1.fq and read_2.fq are pairing. In each file there were about 0.6 million reads and all reads were the same in length. Each pair should belong to the same reference gene and the paired sequences reversed complementary to each other. File read_1 and file read_2 are corresponding to each other in lines. read_1 is positive sequencing result while read_2 is reverse complementary sequencing result and they could be assembled into one tag if both reads were of high quality (Figure 2). Usually raw reads that only have 3′ adaptor fragments should be removed before data analysis. The following analysis was carried out after the dirty raw reads were removed (Illumina report).

### 2.3. Assembly and Alignment

Theoretically, the overlap part of two assembled reads should have totally consistent code. But because the sequencing techniques still have read errors, there will be some low quality locus at the end of the sequence. Generally, when we intend to map reads to reference, we will take a reads quality inspection and cut some length to control the read quality. In this study, to avoid the influence of the final SNP sites statistic caused by such case, we set such locus of each assemble sequence as “N” (Figure 2). In the following basic group frequency statistic of reference sequence, “N” is not participated in the statistic. Thus it eliminates the problem of bad quality of reads in the end; meanwhile it reduces the influence of the SNP quality sites caused by the whole segment sequencing.

As there was no genome reference in nonmodel plant, people usually do mapping works without a genome reference and then calculate the SNPs [11, 12]. Here the DNA sequences of known functional gene were used as reference. To make reads align to reference, we make all the assembled reads into databases with* standalone BLAST tool* (NCBI). Meanwhile to compare the quality difference between assembled reads and nonassembled reads from the same sequence file, among the rest of reads the nonassembled ones were also made into a new database. Then we used the function genes as the query sequence to blast in the database by basic local alignment algorithm [13]. In some of our function genes there are several low-complexity fragments and at the same time the BLAST tool will not calculate the low-complexity part as default. Therefore, we should set the “-F” as “F” to close the low-complexity filter when we use the* blast all* command. To compare the quality of the assembled reads and nonassembled reads, another database was set up by nonassembled reads and the 16 function genes were blast in each database. Blast of 16 genes (with 800 bp average length) in one database containing 0.4 million reads could be completed in 10 minutes by regular PC.

### 2.4. SNPs Calling

Researchers selected SNPs when the MAF is more than 1% for human sequences, while they selected MAF > 5% for plant sequences. All of those are an estimate threshold. As we all know, different experiments may have their own errors and the sequence quality is also different when different technology platforms were used. In this study, we present a new way to find a reasonable MAF for each independent experiment. First we selected some stable genes which were already known as comparable samples and sequence with other samples together. Then the ratios of SNPs change by the MAF were calculated. To observe those trends of SNPs rations variation feature better, polynomial equation was applied to fit the curves (theoretically, N-order polynomial can approximate to any nonlinear function). We derived the first-order differential equation of fitting polynomial equation and that is the accelerating equation of initial equation. The stable value of the accelerated curve was the best threshold.

To check the result of SNPs' ratio by this process, the pretrimmed reads and original reads (clean and adapts discarded) were also used to map and screen SNPs. Three kinds of reads data were compared by SNPs' ratio and position. The assembled reads data should have less SNPs than other reads at the same MAF threshold.

## 3. Result and Discussion

### 3.1. Assembled Reads

16 function gene samples were sequenced in one run and 2 fastq files (each file contains 589573 reads) were output. The usage of the methods referred above to assembled reads and 390992 pairs of reads were successfully assembled. The assembled reads rate was about 66.32%. The average length of assembled reads was 155.10, which illustrated that when two reads assembled nearly 50 bp locus will be overlapped. Over 98.56% assembled reads were assembled by reverse complementary reads; meanwhile the 1.5% assembled reads from others may have very low quality. To get accurate result, raw data were reprocessed (Figure 1), and only assembled reads with both forward and reverse complementary reads were selected for accurate sequence. As we checked the sequence data, only 15~20 bp of original reads in the end were of low quality. Thus the low quality segment of the two reads will be aligned to the other reads (Figure 2). If there is any different code at the alignment locus, that locus will be set as “N” and when we align reads to references sequence, “N” will not be calculated. Thus, the problem of low quality segment in the reads will be solved. In blast result of the nonassembled reads database, most contigs are longer than 80 bp; meanwhile when blasting in assembled reads database, there were many short contigs (more or less than 20 bp) aligned to references. We use standalone BLAST tool to blast function genes in local database. To compare the sequence quality of the assembled and nonassembled reads, we made two local databases.

**Figure 3:**
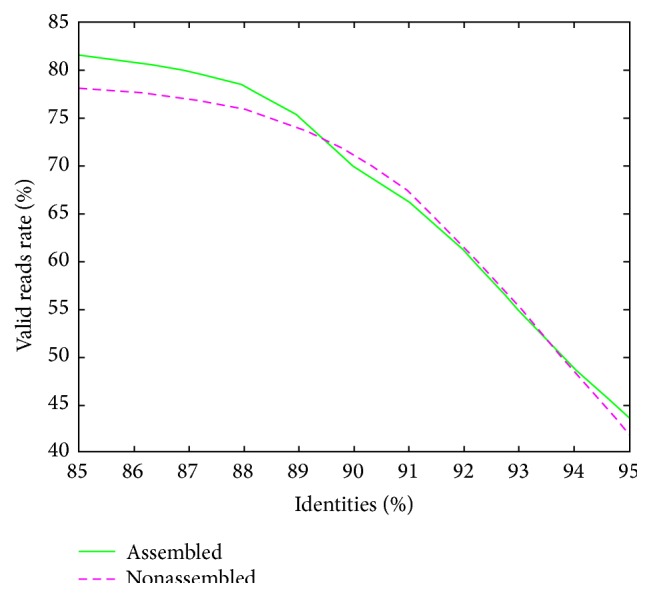
Rate curve of reads can be aligned to reference by identity varied. The valid contigs rate equals the number of the contigs which successfully aligned to references dividing the total reads number in the database.

One database consists of assembled reads and the other consists of nonassembled reads. When blasting in the assembled reads database, 321919 contigs have successfully aligned to the function genes when the identity threshold was set as 85% identities and the number of contigs changed to 249076 by the threshold 90% identities. As a result of blasting in nonassembled database, 314977 contigs from 397162 recorders were aligned to the same query sequence (Table 2). Comparing both assembled and nonassembled valid reads by different blast thresholds, assembled sequence performed high mapping rate (Figure 3). We found that the rates of the successful aligned contigs in each database, both assembled and nonassembled reads, were of little difference. When the identity threshold rises up, the two rate curves become nearly coincident. This illustrated that the assembled reads and nonassembled reads may have similar correct alignment contigs rate on quantity.

### 3.2. SNPs Calling

It is widely believed that SNP could be identified in human gene if the frequency of second base was above 1%. But there is no uniform criterion in plant gene. People who study on crops usually select the threshold value of SNP screening by experience or according to actual condition. If the threshold was too low, some false SNPs would be selected; if the threshold was high, a few of real SNPs could not be completely selected. When threshold was set at 1% in our study, there were too many SNPs in some genes, such as* WCOR14*,* LEA1*, and* LEC1*; the rate of SNP in each reference gene almost reached 40%. That is not reasonable.

**Figure 4:**
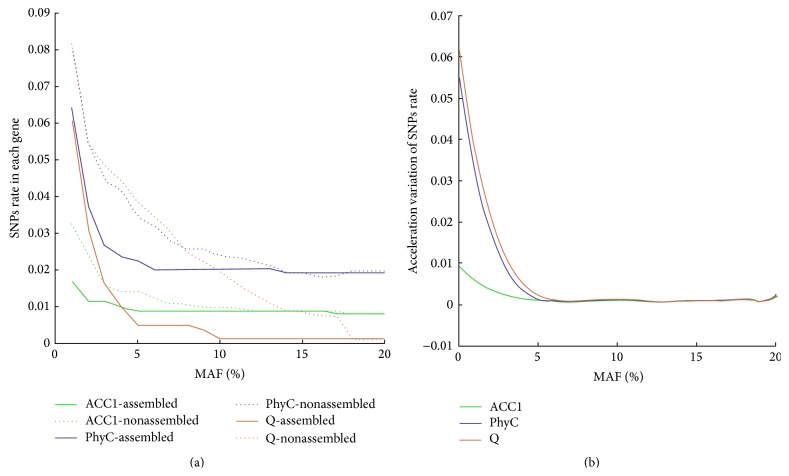
Curve of SNPs rate with the threshold value of MAF variation. (a) SNPs rate curves. The *x*-axis shows the MAF variation and the *y*-axis was the SNPs' proportion in each gene. Solid lines are a result of assembled reads and dotted lines are of nonassembled reads. (b) The curve of accelerating equation from assembled database. The *x*-axis is also the MAF variation, but the *y*-axis was the acceleration of SNPs variation by MAF. The curve was calculated by the fitting polynomial from (a).

To find better threshold value for SNP screening in this study, we set the threshold from 1% to 20% by 1% increase and plotted the curves of* ACC1*,* PhyC*, and* Q* genes' SNP rate variation trend (Figure 4(a)). Because* ACC1*,* PhyC*, and* Q* genes were known as stable genes, we use their SNPs rate variation curve as reference to analysis curve character and find out the best second code rate threshold. We could clearly find that the SNPs rate by nonassembled reads was higher than SNPs rate by assembled reads. Meanwhile the SNPs rate curve of nonassembled keeps descending most of the time, but the assembled reads SNPs curve becomes relatively stable when the second code rises up to 4%. It revealed that nonassembled reads will bring in more SNPs. Most contigs aligned to the function genes in nonassembled reads database were over 80 bp with some low quality locus at the end of the sequence. But the assembled reads have about 50 bp overlap locus at average. When two reads assembled into a sequence, those overlap loci with different codes from two reads were set as “N” (Figure 2) and will not be statistic as potential SNPs. That is why assembled reads are of higher quality than nonassembled reads. So we suggest using assembled sequence to get accurate SNPs or MSV information.

In our study, to find the best MAF to screen SNPs, we only discuss the results from assembled reads database. All the solid lines descended fast in the beginning and become stable after 4% or 5% second code rate (Figure 4). The severe decline may be caused by the sequencing precision. To eliminate the problem by sequencing quality reasonably, selecting an appropriate threshold is more significant. Polynomial fitting method was used to fit the curve to get more information about the curve variation rate. After examination, the 6-order polynomial turned out to be the best one to fit the curves. Then we computed first-order differential of the fitted equation and got the curve variation equations. From derivation equation curve (Figure 4), it showed us the acceleration of SNPs rate descent. When the acceleration became near 0, there were few variations in the initial curve. It means that the rate of SNPs will remain unchanged when the threshold rises up. According to Figure 4, we chose 6% as the second threshold in our study. In future research, the new MAF threshold should be calculated based on the new sequence result.

**Figure 5:**
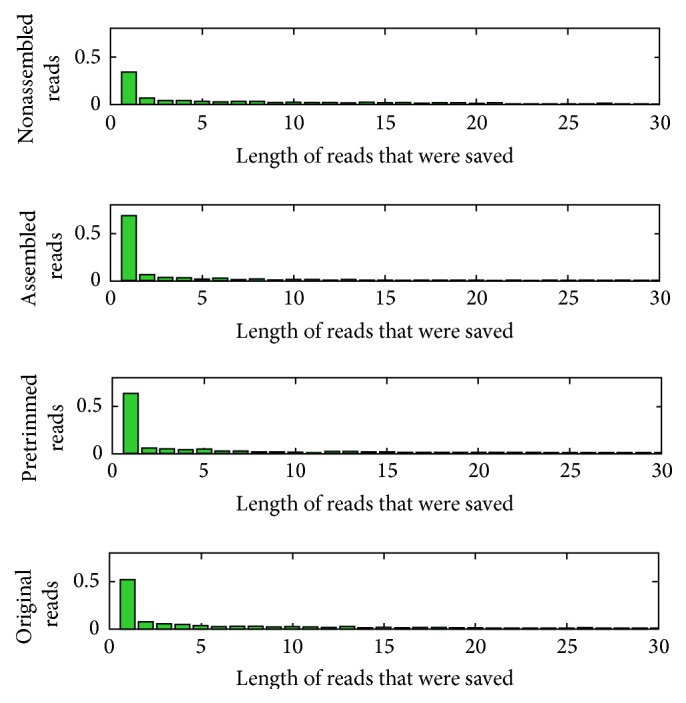
Proportions of reads were trimmed by different length. The *x*-axis was the lengths of reads which were trimmed by local blast algorithm. The *y*-axis was the proportion of each trimmed length. The less the length was trimmed the less the low quality parts the reads have.

As designed, the assembled reads have high quality and when they are aligned to reference genes, they will perform more quality than others reads. Here we compared the castoff length while reads aligned to sequence with nonassembled reads, assembled reads, pretrimmed reads, and original reads. The pretrimmed reads were original reads cut by the end of 20 bp before being used to align to reference. Original reads came from the sequence result without any process. It declared that most reads were zero-cut in the process of alignment (Figure 5). But the assembled reads have more proportion of zero-cut; over 65% reads were zero-cut. Obviously the nonassembled reads have the longest length cut than the other three reads, which illustrated that the reads that cannot be assembled from original reads were of lower quality than the reads that can be assembled. Consequently, if we just use the part of assembled reads for SNPs, we could get more accurate result.

**Figure 6:**
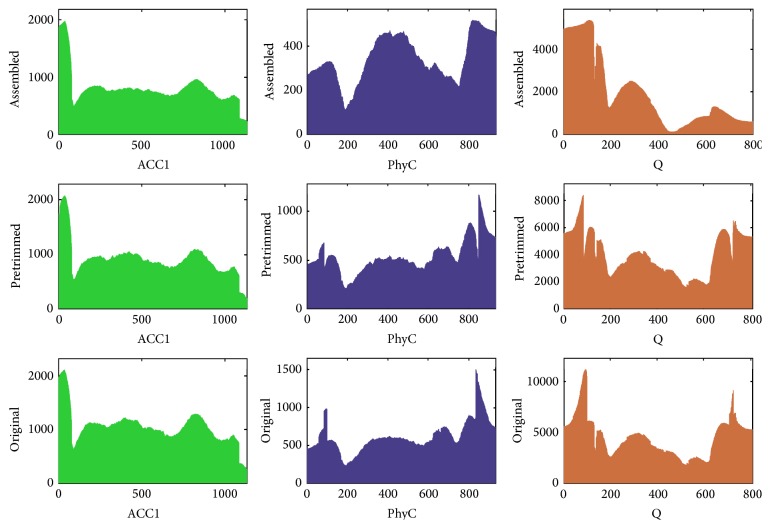
Bar chart of genes locus overlaps by contigs mapping. In each subgraph, the *x*-axis was the whole function gene locus; the *y*-axis was the total number of contigs on each locus.

There are not as much reads as pretrimmed and original reads in assembled database. The overlaps of each gene from assembled reads were lower than other two databases (Figure 6). But in assembled reads database the lowest overlap in* Q* gene still exceeds 100. Although the number of assembled reads is not as much as others, it still has a reliable overlap. We can see that the average overlap of each gene is not homogeneous;* PhyC* gene had 341.83 overlaps,* ACC1* gene 793.03, and* Q* gene 1764.03. That is because the PCR samples concentration we mixed was not under the same uniformity. To get more average overlap, the sample concentration should be as equal as possible.

**Figure 7:**
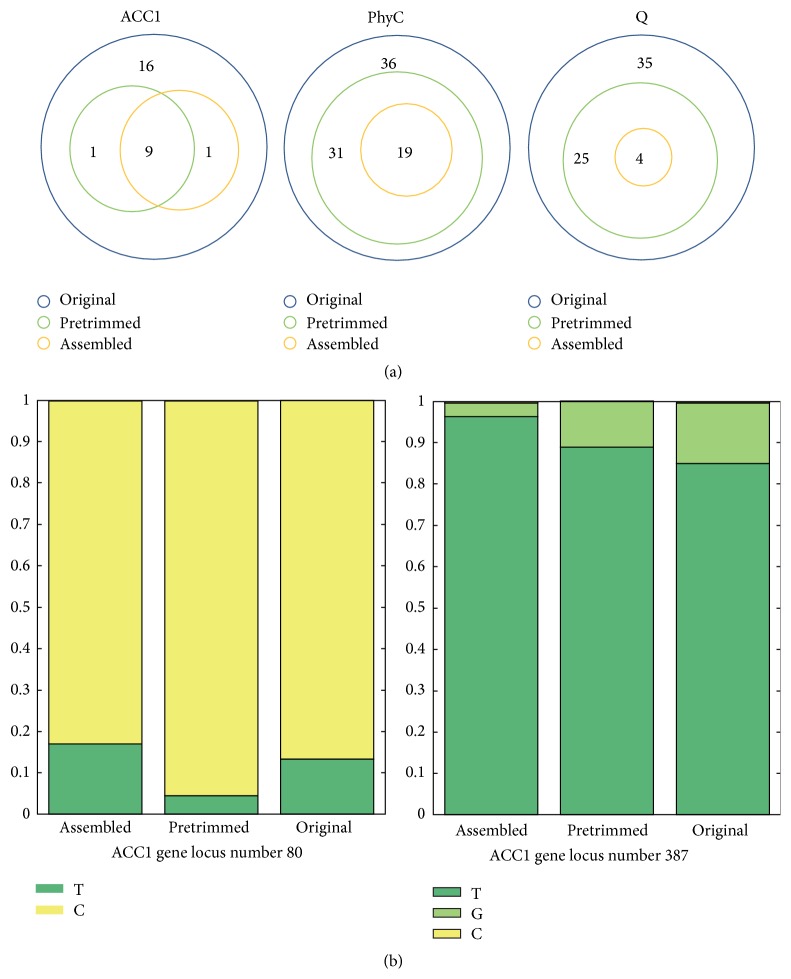
Relationship diagram of SNPs from different reads mapping. (a) The relationship of the SNPs calculated by different data in each gene. (b) The base frequency on mismatch SNPs locus. Different color means different code. The *y*-axis was the proportion.

The advantage of assembled reads in SNPs analysis is that they perform more accurately. In Table 3, there were SNPs from the main stable genes we discussed before. By the same MAF threshold (6%),* ACC1* gene had 10 SNPs from assembled and pretrimmed reads database and had 16 SNPs when aligned by original reads, but in* PhyC* and* Q* gene, less SNPs were screened by assembly. The quality of reads will determine the reliability of SNPs. As original reads have low sequence quality at the end of 15 bp, the pretrimmed reads will surely have high sequence quality and alignment quality. The high-quality reads could avoid bringing too much false SNPs and be aligned to reference more accurate. The SNPs of each gene screened by pretrimmed reads and assembled reads were all overlapped with SNPs from original reads (Figure 7(a)). It is as estimated that assembled and pretrimmed reads will screen less SNPs than original reads. Form the SNPs relationship diagram we can find that most SNPs in assembled reads were overlapped with pretrimmed reads. Only one SNP of* ACC1* gene was not matched. Then we checked that the unmatched SNPs were at 80th (assembled) and 387th (pretrimmed) loci. At the 80th locus, main code was C and minor one is T. The proportion of T from assembled reads was more than that from both original and pretrimmed (Figure 7(b)). Judging from the result of sequencing, different reads had different sequence quality at the same locus, which caused gravity of code skewing to main code. But we set the mismatched locus as “N” without considering the gravity of code when we assembled reads. In that way, the skewing of main code gravity whose low sequence reads brought in was relieved and allowed us to use high-quality reads to get accurate SNPs. At the 387th locus, the proportion of minor code decreased progressively from original to assembled reads. Based on our design ideas, the decrease of minor code proportion may be caused by high-quality reads which we used to align to reference.

**Figure 8:**
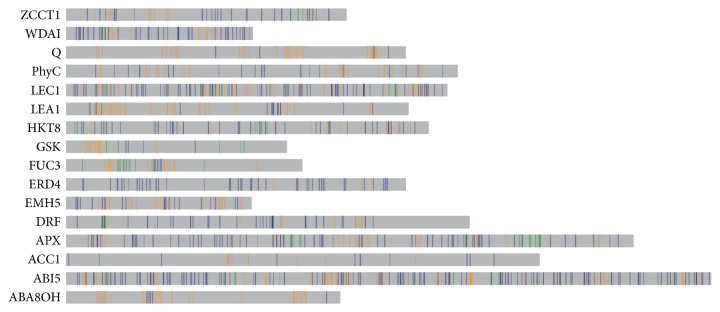
The position of SNPs on the gene. Comparison of SNPs position of the assembled reads and nonassembled reads. The vertical bars were the potential SNPs locus. The green bars form assembled reads, the orange bars form nonassembled reads, and the blue bars belonged to both assembled and nonassembled reads.

We marked all the SNPs from the assembled and nonassembled reads on the genes (Figure 8). There was large amount of distributed SNPs which only discovered in nonassembled reads (orange color) even in stable genes* ACC1*,* PhyC*, and* Q*. Many of them may be false SNPs because of the low quality reads. SNPs markers only from assembled reads (green color) were less than those from nonassembled. It was proved that the reads with higher quality could be assembled easier than that without enough quality. We suggest discarding the reads that could not be assembled when using this method to mine SNPs for getting more reliable information.

The blue and green markers were the final SNPs position tags we found in this study. There were incredible quantities of SNPs in some genes (Figure 8). As wheat was one of organics which have the most complex genome, it has a large genome size and a high proportion of repetitive elements (85–90%) [14, 15]. Many duplicate SNPs may be nothing more than paralogous sequence variants (PSVs). Alternatively, gene conversion in duplicate may generate allelic diversity. So the SNPs in our result could be explained as the PSVs or polymorphism multisite variation (MSV) [16, 17].

## 4. Conclusion

As the high throughput next-generation sequence technology is progressing almost every year, more long read sequence will be brought to us, such as PacBio that will make more easy way for calling SNPs in nonreference species [18]. Particularly for plants with large and complex genome, more long and accurate technology will be helpful in calling SNP [19, 20] (what a pity that PacBio is still a very high-cost way compared to Illumina system). This study aims at finding an efficient and flexible pipeline to mine SNPs with low cost for function genes of nonmodel plant. In outline, our strategy is to mix as much DNA samples as we required and sequence by one run and then use assembled reads to make database for mapping by local blast algorithm computational tools and meanwhile utilize function gene sequence as reference and finally analyze the resulting genotyping data and screen SNPs. The result demonstrated that several function genes of nonmodel plants can be molecular-cloned, mixed to sequence, and analyzed after being assembled and aligned. The assembled reads performed more accurately than the trimmed reads when they are aligned to references (functional genes). Utilizing polynomial fitting and differential equation to find the best MAF threshold is more reasonable.

## Figures and Tables

**Table 1:** Information of sixteen functional genes.

Name	NCBI number	Length	Product
*ABA8OH*	[GenBank: AB455560]	654	ABA 8-hydroxylase
*ABI5*	[GenBank: AB238934]	1540	bZip-type transcription factor TaABI5
*ACC1*	[GenBank: EU660901]	1131	Plastid acetyl-CoA carboxylase
*Apx*	[GenBank: AY513261]	1354	Thylakoid ascorbate peroxidase
*DRF*	[GenBank: FJ560492]	963	Dehydration responsive factor 1 variant
*EMH5*	[GenBank: X73228.1]	443	Early-methionine-labeled protein
*ERD4*	[GenBank: AK330512]	810	Transmembrane protein 63B-like
*FUC3*	[GenBank: BQ806797]	564	Predicted protein
*GSK*	[GenBank: DQ678922]	527	GSK-like kinase 1A
*HKT8*	[GenBank: DQ646339]	866	High affinity K_+_ transporters
*LEA1*	[GenBank: AY148490]	816	Late embryogenesis abundant protein
*LEC1*	[GenBank: BT009029]	910	Nuclear transcription factor Y subunit B1
*PhyC*	[GenBank: AJ295224]	934	Phytochrome C
*Q*	[GenBank: AY702960]	809	Floral homeotic protein
*WDAI*	[GenBank: AY729672]	446	Dimeric alpha-amylase inhibitor
*ZCCT1*	[GenBank: AY485644]	669	Zinc finger-CCT domain

**Table 2:** Elementary information about the reads.

		Reads number	Average length
Assembled	Original reads	390992 (pair)	155.10
	Aligned to reference	219433 (pair)	156.90

Nonassembled	Original reads	198581 (pair)	100
	Aligned to reference	206362 (single)	81.99

The threshold of the aligned identities was 85%.

**Table 3:** The sequence variation information of functional gene by 6% MAF.

Name	Length	Assembled reads		Nonassembled reads		Pretrimmed reads		Original reads	
		Number	Ratio	Number	Ratio	Number	Ratio	Number	Ratio
*ABA8OH*	654	5	0.76%	19	2.91%	13	1.99%	20	3.06%
*ABI5*	1540	159	10.32%	171	11.10%	145	9.42%	152	9.87%
*ACC1*	1131	10	0.88%	14	1.24%	10	0.88%	16	1.41%
*APX*	1354	97	7.16%	86	6.35%	83	6.13%	96	7.09%
*DRF*	963	42	4.36%	39	4.05%	41	4.26%	46	4.78%
*EMH5*	443	21	4.74%	29	6.55%	20	4.51%	27	6.09%
*ERD4*	810	46	5.68%	44	5.43%	42	5.19%	45	5.56%
*FUC3*	564	18	3.19%	13	2.30%	20	3.55%	22	3.90%
*GSK*	527	9	1.71%	9	1.71%	15	2.85%	20	3.80%
*HKT8*	866	51	5.89%	36	4.16%	44	5.08%	56	6.47%
*LEA1*	816	14	1.72%	44	5.39%	44	5.39%	61	7.48%
*LEC1*	910	92	10.11%	90	9.89%	83	9.12%	110	12.09%
*PhyC*	934	19	2.03%	30	3.21%	31	3.32%	36	4.07%
*Q*	809	4	0.49%	28	3.46%	25	3.09%	35	4.20%
*WDAI*	446	44	9.87%	47	10.54%	38	8.52%	34	7.62%
*ZCCT1*	669	28	4.19%	34	5.08%	21	3.14%	28	4.19%
